# Safety and tolerability of astegolimab, an anti-ST2 monoclonal antibody: a narrative review

**DOI:** 10.1186/s12931-025-03360-0

**Published:** 2025-10-29

**Authors:** Steven G. Kelsen, Marcus Maurer, Michael Waters, Ajit Dash, Alice Fong, Divya Mohan, Wiebke Theess, Xiaoying Yang, Giuseppe Alvaro, Christopher E. Brightling

**Affiliations:** 1 Lewis Katz School of Medicine at Temple University, Philadelphia, PA USA; 2 Institute of Allergology, Charité – Universitätsmedizin Berlin, corporate member of Freie Universität Berlin and Humboldt-Universität zu Berlin, Berlin, Germany; 3 Fraunhofer Institute for Translational Medicine and Pharmacology ITMP, Immunology and Allergology, Berlin, Germany; 4 Velocity Clinical Research, Chula Vista, CA USA; 5 Genentech, Inc., South San Francisco, CA USA; 6 F. Hoffmann-La Roche, Ltd., Basel, Switzerland; 7 Institute for Lung Health, National Institute for Health and Care Research, Leicester Biomedical Research Centre, University of Leicester, Leicester, UK

**Keywords:** Astegolimab, Asthma, Chronic obstructive pulmonary disease, Interleukin-33 (IL-33), Infection, Inflammation, Immunogenicity, Major adverse cardiac events, Safety, ST2

## Abstract

Chronic inflammation is an underlying feature of respiratory diseases such as chronic obstructive pulmonary disease (COPD). Novel therapies that target the inflammatory mechanisms driving acute exacerbations of COPD are required. The ST2 receptor, which binds the alarmin interleukin (IL)-33 to initiate an inflammatory response, is a potential target. Astegolimab, a fully human immunoglobulin G2 monoclonal antibody, which binds with high affinity to ST2 to prevent binding of IL-33, is a potential therapy for COPD. However, targeting inflammatory pathways that form part of the immune system may have unintended consequences, such as implications for the response to infection and cardiovascular function. Therefore, an understanding of astegolimab’s safety profile in clinical use is essential. This narrative review summarizes clinical safety data from published clinical trials of astegolimab with a focus on adverse events of interest, including infections and cardiac events. Astegolimab was shown to be well tolerated in > 580 patients with asthma, atopic dermatitis, COPD, and severe COVID-19 pneumonia who took part in Phase II trials. The frequency of adverse events (AEs) and serious AEs was similar between the astegolimab and placebo arms in each trial (AEs: 41–81% vs. 58–77%; serious AEs: 3–29% vs. 0–41%, respectively). The number of deaths was similar between treatment arms and there were no astegolimab-related deaths. Astegolimab did not increase the risk of infection or major adverse cardiac events. Ongoing Phase IIb and Phase III trials of astegolimab in patients with COPD who have a history of frequent acute exacerbation(s) of COPD will provide a future opportunity to confirm the safety profile of astegolimab.

**Supplementary Information:**

The online version contains supplementary material available at 10.1186/s12931-025-03360-0.

## Background

Chronic inflammation is characteristic of airway diseases such as chronic obstructive pulmonary disease (COPD) and asthma [1–4]. Acute exacerbations of COPD (AECOPD) are common, characterized by the acute worsening of symptoms that are associated with localized and systemic inflammation, and triggered by bacterial or viral airway infections, tobacco smoke, or environmental factors [3, 5–10]. The consequences of AECOPD include worsening of lung function and quality of life, increased mortality, and a substantial economic burden [5, 11–13]. Novel therapeutic approaches that target the underlying inflammatory mechanisms of AECOPD are, therefore, urgently needed [14].

Targeting the ST2 receptor (IL1RL1), which is expressed on a variety of inflammatory and immune-related cells [15, 16], is one such approach. ST2 binds the alarmin interleukin (IL)-33 and subsequently forms a transmembrane heterodimer with the IL-1 receptor accessory protein (IL1RAP) to initiate downstream innate and adaptive immune inflammatory responses [15, 17]. ST2-activated cells release cytokines that promote both type-1 (neutrophilic) and type-2 (eosinophilic) inflammation [15].

Astegolimab is a fully human immunoglobulin G2 monoclonal antibody that binds with high affinity to ST2, thereby preventing the binding of IL-33 and its downstream signaling [18]. In COPD, it is proposed that astegolimab blocks the initiation of inflammation driven by ST2/IL-33 binding to prevent the onset of AECOPD. Astegolimab efficacy in respiratory disease has been evaluated in Phase II trials. In the ZENYATTA trial (NCT02918019; 27 September 2016), 502 patients with severe asthma treated with astegolimab had a statistically significant 43% reduction in asthma exacerbations over 52 weeks relative to placebo (*P* = 0.0049) [18]. The COPD-ST2OP trial (NCT03615040; 4 May 2018) in 81 patients with moderate-to-very-severe COPD, showed a clinically meaningful reduction in the annual exacerbation rate of 22% for astegolimab versus placebo, though this result did not reach statistical significance [19].

Biologic therapies that act upon immune-related pathways may have effects beyond inhibition of the disease-associated inflammation [20]. For example, inhibiting ST2/IL-33 binding on macrophages may impede mucosal healing and wound repair in inflammatory bowel disease [21]. Furthermore, inhibition of the ST2/IL-33 pathway and subsequent dampening of the downstream MyD88/MAPK/NF-κB inflammatory pathways may prevent the secretion of cytokines from immune cells, potentially impacting infection response [22]. Inflammation is an important defense against infection, and the ST2/IL-33 pathway plays a pivotal role in the response to microbial infections following the release of IL-33 upon exposure to viruses, bacteria, fungi, or parasites [22]. However, preclinical studies have shown that the role of ST2/IL-33 can be either protective or detrimental, depending on the specific infection [22–28]. For example, mortality due to *Streptococcus pyogenes* infection was increased in ST2 and IL-33 knock-out mice compared with wild-type [23] and ST2 was shown to be required for efficient infection control during mouse cytomegalovirus infection [26]. Conversely, mice lacking ST2 had greater resistance to *Leishmania infantum* infection compared with wild-type mice [27], and ST2 knock-out mice infected with *Plasmodium chabaudi* had improved survival compared with wild-type mice [28]. Considering the inconsistent preclinical picture, it is therefore plausible that blocking the ST2/IL-33 pathway may impede the appropriate inflammatory responses to infections in clinical settings.

Furthermore, the ST2/IL-33 pathway has been proposed to have potential involvement in the cardiac system; however, due to conflicting preclinical evidence, it is not clear if ST2 has a protective or detrimental role in cardiovascular function [29–31]. For example, ST2 knock-out mice had cardiac hypertrophy and fibrosis following transverse aortic constriction, suggesting a cardioprotective role of ST2 in mechanical overload [29]. On the contrary, ST2 and IL-33 have been found in human atherosclerotic tissues where they stimulated the production of adhesion molecules and chemokines [30], although a preclinical study found no role of ST2/IL-33 in the development of atherosclerosis in ApoE^−/−^ mice [31]. Activation of the ST2/IL-33 pathway may also contribute to atrial arrythmia and fibrillation [32]. Moreover, in a chronic heart transplantation rejection mouse model, ST2 deficiency played both protective and exacerbating roles in cardiac allograft vasculopathy depending on whether the ST2 deficiency was in the donor or recipient, respectively [33]. With this mixed preclinical evidence regarding the role of ST2 in infection and cardiovascular function, there remains a hypothetical link between loss of function of ST2 and infection and cardiovascular risk that requires further clinical data to determine impact.

This review evaluates the safety data from published clinical trials of astegolimab to establish its safety profile, with a particular focus on aspects that have been identified as potential risks with ST2/IL-33 blockade.

## Methods

PubMed was searched up to and including 25 March 2024 for the following terms in the title or abstract: ‘astegolimab’; ‘MSTT1041A’; ‘RO7187807’. The search returned 10 peer-reviewed journal publications. Following the exclusion of reviews (*n* = 2), comments (*n* = 1), and research articles not reporting safety data (*n* = 2), a total of five primary publications were included in this review of astegolimab safety data. Due to differences in study designs and populations, no additional statistical analyses or pooling of data were performed and all comparisons made in this review between treatment arms or trials are descriptive in nature.

Adverse events (AEs) of interest for this review included: infections; MACE; infusion-related reactions, anaphylaxis, anaphylactoid reactions, and hypersensitivity reactions; and injection site reactions. AEs of infections were those listed under the MedDRA System Organ Classes ‘Infections and Infestations’. MACE were defined in the ZENYATTA, ZARNIE (NCT03747575; 16 November 2018), and COPD-ST2OP trials as death due to cardiovascular causes; non-fatal myocardial infarction; non-fatal stroke or transient ischemic attack; unstable angina or chest pain requiring hospitalization; coronary revascularization; and congestive heart failure requiring hospitalization. In the COVASTIL trial (NCT04386616; 11 May 2020), the MACE definition was adapted to encompass death due to primary cardiovascular causes, non-fatal myocardial infarction or acute coronary syndrome, and new or worsening heart failure. Limited MACE data have been published for COVASTIL [34]; however, additional data for COVASTIL and the other Phase II trials were available on file, and are reported here to provide a detailed overview of MACE with astegolimab.

## Published randomized, double-blind, placebo-controlled trials of astegolimab

The publications included in this review reported safety data from four Phase II randomized, double-blind, placebo-controlled trials of astegolimab in inflammatory conditions: severe asthma (ZENYATTA [18]), moderate-to-severe atopic dermatitis (ZARNIE [35]), moderate-to-very-severe COPD (COPD-ST2OP [19]) and patients hospitalized with severe COVID-19 pneumonia (COVASTIL [34]). Safety data were available for a total of 581 patients treated with astegolimab and 331 patients treated with placebo. The fifth publication included selected immunogenicity data from a Phase I trial of astegolimab [36].

**Fig. 1:**
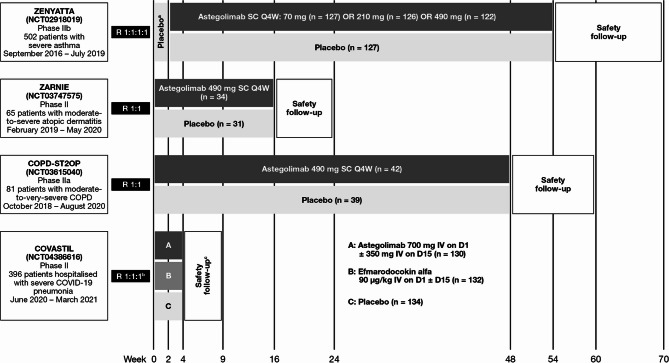
An overview of published randomized, double-blind, placebo-controlled Phase II/IIa/IIb trials of astegolimab. For each trial, patient numbers reflect the safety evaluable populations. ^a^Placebo run-in period. ^b^Patients in COVASTIL were randomized to receive astegolimab, efmarodocokin alfa, or placebo at a 1:1:1 ratio; data from the efmarodocokin alfa arm are not reported in this manuscript. ^c^Patients were followed until Day 60. COPD, chronic obstructive pulmonary disease; COVID-19, coronavirus disease 2019; D, day; IV, intravenous; Q4W, every 4 weeks; R, randomized; SC, subcutaneous

An overview of the Phase II trial designs is provided in Fig. 1. Astegolimab was administered subcutaneously in ZENYATTA, ZARNIE, and COPD-ST2OP, with doses ranging from 70 to 490 mg every 4 weeks for between 16 and 52 weeks [18, 19, 35]. In COVASTIL, in which patients had severe COVID-19 pneumonia, astegolimab was administered intravenously as a single dose of 700 mg, with a second dose (350 mg) administered 2 weeks later if the patient remained hospitalized and on supplemental oxygen [34]. A second active-treatment arm in COVASTIL evaluated the human IL-22 immunoglobulin G4 fusion protein efmarodocokin alfa [34]; this treatment arm is not discussed further in this review.

## Overview of safety data

A summary of AEs is reported in Table 1. The overall AE incidence in ZENYATTA, COPD-ST2OP, and COVASTIL was generally similar between treatment arms [18, 19, 34] and was higher for placebo than astegolimab in ZARNIE [35]. The incidence of serious AEs and deaths was generally similar between treatment arms in all trials and no astegolimab-related deaths were reported [18, 19, 34, 35]. Most deaths (*n* = 46/50; 92%) occurred in COVASTIL (mostly attributed to COVID-19 pneumonia, respiratory failure, multiple organ system failure, or sepsis) and occurred at the same frequency in the astegolimab and placebo arms [34]. The most common AEs are summarized in Supplementary Table S1, and there were no trends towards specific AEs [18, 19, 34, 35]. There were no clinically meaningful changes across treatment arms in laboratory parameters, vital signs, or ECGs in ZENYATTA or ZARNIE (data were not published for COPD-ST2OP or COVASTIL) [18, 35].

**Table 1:** Overview of AEs reported in Phase II clinical trials of astegolimab

	ZENYATTA (severe asthma) [18]		ZARNIE (moderate-to-severe atopic dermatitis) [35]		COPD-ST2OP (moderate-to-very-severe COPD) [19]		COVASTIL (severe COVID-19 pneumonia) [34]	
	Astegolimab pooled^a^ (*n* = 375)	Placebo (*n* = 127)	Astegolimab 490 mg SC Q4W (*n* = 34)	Placebo (*n* = 31)	Astegolimab 490 mg SC Q4W (*n* = 42)	Placebo (*n* = 39)	Astegolimab 700 mg IV on D1 ± 350 mg IV on D15 (*n* = 130)	Placebo (*n* = 134)
Deaths, n (%)	2 (1)	0	0	0	0	2 (5)	23 (17)	23 (17)
Patients who withdrew from trial due to an AE, n (%)	0	0	0	0	NR	NR	1 (1)	1 (1)
Patients with any AE,^b^ n (%)	269 (72)	98 (77)	14 (41)	18 (58)	34 (81)	28 (72)	85 (65)	87 (65)
Serious AE	29 (8)	8 (6)	1 (3)^c^	0	12 (29)	16 (41)	38 (29)	38 (28)
Related AE	32 (9)	4 (3)	2 (6)	5 (16)	NR	NR	12 (9)	15 (11)
AEs of interest, n (%)								
Infection and/or infestation	168 (45)	65 (51)	5 (15)	10 (32)	NR^d^	NR^d^	31 (23)^e^	34 (26)^e^
Potential MACE^f^	2 (1)	1 (1)	0	0	0	1 (3)	4 (3)	2 (1)
Infusion-related reaction,^g^ anaphylaxis, anaphylactoid, and hypersensitivity reaction	1 (<1)	1 (1)	0	0	0	0	0^h^	2 (1)^h^
Injection site reaction	24 (6)^i^	1 (1)	0	1 (3)	1 (2)^h^	4 (10)^g^	NA^j^	NA^j^

^a^Pooled data for patients receiving astegolimab SC 70 mg Q4W (*n* = 127), 210 mg Q4W (*n* = 126), or 490 mg Q4W (*n* = 122)

^b^Most AEs in ZENYATTA and all AEs in ZARNIE were mild or moderate in severity [18, 35]. Of the patients in COVASTIL who had ≥ 1 AE, 43/87 (49%) receiving placebo and 46/85 (54%) receiving astegolimab experienced ≥ 1 AE that was Grade ≥ 3 [34]. AE severity was not reported for COPD-ST2OP

^c^One patient in the astegolimab group experienced a serious AE of aortic aneurysm. This was a worsening of a pre-existing condition and considered unrelated to study treatment

^d^For COPD-ST2OP, the number of patients who experienced infections and infestations was not reported. However, the total number of infections/infestations across all AEs was reported as 20/122 AEs (16%) in the astegolimab arm and 20/100 AEs (20%) in the placebo arm

^e^Data on file; serious AEs of infection were previously reported for COVASTIL: placebo, *n* = 19 (14%); astegolimab, *n* = 18 (14%) [34]

^f^Total number of MACE in COVASTIL was previously reported [34]; all other MACE data are data on file

^g^Infusion-related reactions applicable to COVASTIL only

^h^Data on file

^i^No dose-dependent response for injection site reactions was observed: astegolimab 70 mg, *n* = 10 (8%); astegolimab 210 mg, *n* = 8 (6%); astegolimab 490 mg, *n* = 6 (5%)

^j^Patients in the COVASTIL trial received treatment via IV infusion.

*AE* adverse event, *COPD* chronic obstructive pulmonary disease, *COVID-19* coronavirus disease 2019, *D* day, *IV* intravenous, *MACE* major adverse cardiac event, *NA* not applicable, *NR* not reported, *Q4W* every 4 weeks, *SC* subcutaneous

## Adverse events of interest

### Infections

Infections occurred in 15–45% of patients treated with astegolimab and 26–51% of patients treated with placebo, and there was no increase in rates of infections following astegolimab treatment compared with placebo (Table 1) [18, 19, 34, 35]. Importantly, no dose-dependent incidence of infections was observed in ZENYATTA (placebo, *n* = 65 [51.2%]; astegolimab 70 mg, *n* = 55 [43.3%]; astegolimab 210 mg, *n* = 58 [46.0%]; astegolimab 490 mg, *n* = 55 [45.1%]) [18].

Nasopharyngitis was the most frequently reported infection in ZENYATTA (all patients, 13%; astegolimab, *n* = 50 [13%]; placebo, *n* = 14 [11%]) and ZARNIE (all patients, 5%; astegolimab, *n* = 0 [0%]; placebo, *n* = 3 [10%]) [18, 35]. Furthermore, there was no difference in serious AEs of infection between treatment arms in ZENYATTA (pneumonia: placebo, *n* = 1 [1%], astegolimab, *n* = 2 [1%]; pyelonephritis: placebo, *n* = 1 [1%], astegolimab, *n* = 1 [< 1%]) [18]. All infections in ZARNIE were non-serious [35]. In COPD-ST2OP, 20 AEs were reported per treatment arm, including upper respiratory tract infection, lower respiratory tract infection, urinary tract infections, and cellulitis [19]. The most common infection in COVASTIL, aside from COVID-19 and/or pneumonia, was urinary tract infection (placebo, *n* = 4 [3%], astegolimab, *n* = 1 [1%]), and the most common serious infection, apart from COVID-19 and/or pneumonia, was septic shock (placebo, *n* = 3 [2%], astegolimab, *n* = 3 [2%]) [34].

### Major adverse cardiac events

The incidence of potential MACE in the astegolimab and placebo arms of each trial ranged from 0 to 3% (Table 1; data on file). Overall, the incidence of potential MACE in each trial was similar in the placebo and astegolimab arms (1% in each arm). No MACE were considered related to astegolimab.

A breakdown of MACE is provided in Supplementary Table S2. In ZENYATTA, the three MACE were atrial tachycardia, chest pain, and atrial fibrillation, none of which led to treatment discontinuation, and all resolved (data on file). No MACE were reported in ZARNIE, and one MACE of heart failure was reported in COPD-ST2OP (data on file). In COVASTIL, more MACE were reported in astegolimab-treated patients (3%) than in placebo-treated patients (1%) [34]; however, further analysis within the system organ class of cardiac disorders showed no significant imbalance between the two arms [34].

### Infusion-related reactions, anaphylaxis, anaphylactoid reactions, and hypersensitivity reactions

The incidence of infusion-related reactions, anaphylaxis, anaphylactoid reactions, and hypersensitivity reactions is shown in Table 1, and further details are given in Supplementary Table S2. In ZENYATTA, the moderate AE of hypersensitivity occurred within 24 h of astegolimab administration in Week 34 in a patient with a history of drug hypersensitivity reaction and was considered related to astegolimab; the AE resolved 5 days after onset, following astegolimab withdrawal (data on file). The severe serious AE of anaphylaxis occurred 10 days after administration of the second dose of placebo and resolved 6 days after onset without dose adjustment (data on file). In COVASTIL, there were two infusion-related reactions of tachycardia and respiratory distress in the placebo arm (data on file).

### Injection site reactions

Injection site reactions occurred in 0–6% of patients receiving astegolimab and 1–10% of patients receiving placebo (Table 1). Injection site reactions were the most common treatment-related AE in ZENYATTA and occurred more frequently in the astegolimab arm than the placebo arm [18]; this contrasts with ZARNIE and COPD-ST2OP, in which injection site reactions were more common in the placebo arm than in the astegolimab arm [35] (data on file). No dose-dependent response was observed in ZENYATTA (astegolimab 70 mg, *n* = 10 [8%]; astegolimab 210 mg, *n* = 8 [6%]; astegolimab 490 mg, *n* = 6 [5%]) [18]. All injection site reactions in ZENYATTA and ZARNIE were non-serious [18, 35].

## Immunogenicity

The incidence of treatment-induced anti-astegolimab ADAs in the astegolimab arms ranged from 0 to 7% (Table 2) [18, 34, 35]. There was no effect of ADAs on astegolimab pharmacokinetics, efficacy, or safety in ZENYATTA and COVASTIL, and there was no dose-dependent effect on ADA formation in the ZENYATTA trial [18, 34]. In an analysis of single-dose subcutaneous astegolimab in healthy volunteers in a Phase I study, ADAs occurred in up to 33% of participants in selected cohorts, but had no significant impact on the pharmacokinetics of astegolimab [36].

**Table 2:** Incidence of treatment-induced^a^ anti-astegolimab antibodies during Phase II trials of astegolimab

	ZENYATTA (severe asthma) [18] (*n* = 368)	ZARNIE (moderate-to-severe atopic dermatitis) [35] (*n* = 33)	COPD-ST2OP (moderate-to-very-severe COPD) [19] (*n* = 42)	COVASTIL (severe COVID-19 pneumonia) [34] (*n* = 104)
Binding ADA positive	26 (7)	1 (3)	0^c^	2 (2)
Transient^b^	NR	1 (3)	0^c^	NR
Neutralizing ADA positive	NR	NR	NR	NR
Transient^b^	NR	NR	NR	NR

All values are *n* (%)

^a^Participants who received astegolimab and had a post-baseline result and a negative or no result at baseline

^b^Negative result at the participant’s last timepoint or at subsequent timepoints tested within the study period

^c^One patient in the astegolimab treatment arm had a positive ADA result at baseline that was treatment unaffected, i.e., still positive post-baseline with no significant increase in titers throughout the study

*ADA* anti-drug antibody, *COPD* chronic obstructive pulmonary disease, *COVID-19* coronavirus disease 2019, *NR* not reported

## Discussion

Astegolimab has been shown to be well tolerated in over 580 patients with asthma, atopic dermatitis, COPD, or severe COVID-19 pneumonia during Phase II clinical trials. The frequency of AEs and serious AEs was generally similar between the astegolimab and placebo arms, and there was no difference in the number of deaths between treatment arms, nor were any of the deaths related to astegolimab. Astegolimab trial discontinuations due to AEs were low and balanced between treatment arms.

There was no evidence of astegolimab increasing the risk of infection compared with placebo in any of the four trials, including in the Phase II ZENYATTA and COPD-ST2OP trials, which each had follow-up periods of ≥ 1 year. In all trials, the incidence of infections was lower in the astegolimab arm(s) than the placebo arm, and no astegolimab dose-related response was seen [18, 19, 34, 35]. This finding is important as it has been reported previously that some monoclonal antibodies targeting immune cells and cytokines can increase the risk of infection [37]. Furthermore, many of the patients participating in the astegolimab trials were at increased risk of infection prior to the trial commencing due to their medical condition. For example, atopic dermatitis increases the risk of skin and systemic infections due to skin barrier defects, immune dysregulation, *Staphylococcus aureus* colonization, and dysbiosis of skin flora [38–40]. A systematic review also revealed increased rates of respiratory infections and non-respiratory infections in patients with asthma [41]. The lack of increased susceptibility to infection following astegolimab treatment is therefore an encouraging sign that patients are not experiencing a treatment-related increase in immunosuppression despite having disease-associated predisposition to infection.

The impact of ST2 inhibition on cardiovascular risk was an important safety aspect to evaluate due to a lack of clarity on the involvement of ST2 in cardiac function from the preclinical studies [29–33]. However, there was no evidence of increased risk of MACE with astegolimab compared with placebo in the clinical trials, with events being balanced between treatment arms. This lack of cardiac effect with ST2/IL-33 pathway inhibition observed in the Phase II trials is particularly encouraging given that the patient populations enrolled in these trials (for example, patients with COPD who experience frequent exacerbations and patients with severe COVID-19) are considered to be at risk of cardiac events [42, 43]. The clinical results are consistent with safety pharmacology studies in cynomolgus monkeys, which showed no effects related to astegolimab in any cardiovascular parameters, up to the highest dose tested (data on file). The results are also consistent with previous reports of the IL-33 inhibitor, tozorakimab. In the Phase IIb FRONTIER-1 trial of patients with diabetic kidney disease, the incidences of AEs and serious AEs of cardiac disorders were lower in the tozorakimab arm (3.7% and 1.8%, respectively) than the placebo arm (6.5% and 2.2%, respectively) [44], while in the Phase IIa ACCORD-2 trial in patients hospitalized with COVID-19, AEs of cardiac disorders occurred in 13.0% of patients in the tozorakimab arm and 11.4% in the placebo arm [45]. Together, these data suggest no increased cardiovascular risk associated with inhibiting the ST2/IL-33 pathway in clinical settings across multiple conditions. However, more rigorous assessments of cardiac risk and longer-term studies are needed to confirm these findings. Ongoing trials of astegolimab will continue to monitor MACE to determine if any causal relationship between ST2 and clinical cardiac risk exists.

There was no increased risk of anaphylaxis, anaphylactoid reactions, or hypersensitivity reactions in the astegolimab arm compared with placebo, although it is important to note that patients with a history of severe allergic reaction or anaphylactic reaction to a biologic therapy were excluded from these astegolimab trials. Increased risk of anaphylaxis with some monoclonal antibodies used in the treatment of severe asthma has been reported in two retrospective analyses of real-world data, with omalizumab, benralizumab, and mepolizumab having an association with anaphylaxis in both studies [46, 47]. Dupilumab, on the other hand, showed no association with anaphylaxis [46, 47]. It will be important to continue to monitor for anaphylaxis, anaphylactoid reactions, and hypersensitivity reactions as more data become available in ongoing astegolimab trials.

The incidence of treatment-induced anti-astegolimab ADAs observed in the Phase II trials was lower than that reported previously for Phase I trials of astegolimab in healthy participants, in which 5/35 participants (14%) with subcutaneous dosing and 4/12 (33%) with intravenous dosing developed astegolimab ADAs after single dosing and 5/24 participants (21%) with subcutaneous dosing and 3/6 (50%) with intravenous dosing developed anti-astegolimab antibodies after multiple dosing [48]. The presence of astegolimab ADAs did not impact the pharmacokinetics of astegolimab [36, 48]. The higher ADA incidence in Phase I trials compared with Phase II may be related to multiple factors. For instance, there were differences in assay design between Phase I and II trials, and the Phase I trials were conducted in small populations of healthy volunteers (*n* = 30, 31, and 48), compared with the larger Phase II trials in patients with different diseases. Furthermore, participants in the Phase II trials may have been taking immunosuppressant medications, which could have impacted their immune response. These factors make direct rate comparisons difficult; however, to date, there has been no reported impact of ADAs on safety or drug exposure. A systematic review and meta-analysis of ADA incidence in > 12,300 patients from 43 studies receiving monoclonal antibody treatment for moderate-to-severe asthma found ADAs in 0–8% of patients (omalizumab 0%; tezepelumab, 1%; mepolizumab, 4%; reslizumab, 4%; dupilumab, 8%; benralizumab, 8%) [49], which is broadly in line with the incidence of treatment-induced astegolimab ADAs seen in the Phase II astegolimab trials. The same study reported no meaningful effect of ADAs on efficacy or safety outcomes, but did note a potential dose–response effect on the formation of ADAs for some drugs, with lower or less frequent doses being more likely to induce the formation of ADAs, and indicated that subcutaneous dosing may be more likely to induce ADAs than intravenous administration [49].

The first approved biologics targeting inflammatory pathways in COPD, dupilumab (an anti–IL-4R/IL-13R) and mepolizumab (an anti–IL-5), are indicated for use in adults with inadequately controlled COPD and an eosinophilic phenotype. The overall incidence of AEs, serious AEs, and deaths in Phase III trials were generally similar for both drugs compared with placebo, with no apparent increase in infection rate or cardiac events upon treatment with the biologic [50–52]. Furthermore, additional studies of these drugs in patients with moderate-to-severe or severe asthma showed consistency with known safety profiles and no negative effect on safety following long-term treatment and follow-up (3–~10 years) [53–55]. These approved treatments are well tolerated and provide additional evidence for the feasibility of targeting inflammatory pathways in COPD without negatively impacting infection response or cardiac function [50–55]. When considering the requirement for long-term treatment of chronic conditions, the 52-week treatment period of the ZENYATTA trial provides initial support for the long-term safety and tolerability of astegolimab beyond its use in an acute setting. However, there is a need for future long-term extension studies and real-world evidence to confirm that there are no longer-term AEs that have hitherto not been observed. Additional Phase IIb and Phase III trials of astegolimab are currently ongoing in an estimated 2624 patients with COPD who have a history of frequent AECOPDs (NCT05037929, 31 August 2021; NCT05595642, 24 October 2022; NCT05878769, 19 May 2023). These trials will provide a further opportunity to confirm the safety profile of astegolimab.

## Supplementary Information

Supplementary Material 1.

## Data Availability

Qualified researchers may request access to individual patient-level data through the clinical study data request platform ([https://vivli.org/](https:/vivli.org)). Further details on Roche’s criteria for eligible studies are available here: [https://vivli.org/members/ourmembers/](https:/vivli.org/members/ourmembers). For further details on Roche’s Global Policy on the Sharing of Clinical Information and how to request access to related clinical study documents, see here: [https://www.roche.com/innovation/process/clinical-trials/data-sharing](https:/www.roche.com/innovation/process/clinical-trials/data-sharing).

## Abbreviations

ADA: Anti-drug Antibody
AE: Adverse Event
AECOPD: Acute Exacerbation(s) of COPD
AESI: Adverse Event of Special Interest
COPD: Chronic Obstructive Pulmonary Disease
COVID-19: Coronavirus Disease 2019
D: Day
ECG: Electrocardiogram
IL: Interleukin
IL1RAP: Interleukin-1 Receptor Accessory Protein
IV: Intravenous
MACE: Major Adverse Cardiac Events
MedDRA: Medical Dictionary for Regulatory Activities
NA: Not Applicable
NR: Not Reported
Q4W: Every 4 Weeks
R: Randomized
SC: Subcutaneous
