# Supplementary material for: Safety and tolerability of astegolimab, an anti-ST2 monoclonal antibody: a narrative review
Source: Respir Res. 2025 Oct 29;26:302. doi: 10.1186/s12931-025-03360-0 (PMC12574037; doi:10.1186/s12931-025-03360-0)
Supplement: Supplementary file 1 — Supplementary Material 1. [file 12931_2025_3360_MOESM1_ESM.pdf]

Supplementary material for:

Safety and tolerability of astegolimab, an anti-ST2 monoclonal antibody: a narrative review

Steven G. Kelsen,[1] Marcus Maurer,[^2,3] Michael Waters,[4] Ajit Dash,[5] Alice Fong,[5] Divya Mohan,[5] Wiebke Theess,[6] Xiaoying Yang,[5] Giuseppe Alvaro,[6] Christopher E. Brightling[7]

[1]Lewis Katz School of Medicine at Temple University, Philadelphia, PA, USA; [2]Institute of Allergology, Charité – Universitätsmedizin Berlin, corporate member of Freie Universität Berlin and Humboldt-Universität zu Berlin, Berlin, Germany; [3]Fraunhofer Institute for Translational Medicine and Pharmacology ITMP, Immunology and Allergology, Berlin, Germany; [4]Velocity Clinical Research, Chula Vista, CA, USA;[5]Genentech, Inc., South San Francisco, CA, USA; [6]F. Hoffmann-La Roche, Ltd., Basel, Switzerland;[7]Institute for Lung Health, National Institute for Health and Care Research, Leicester Biomedical Research Centre, University of Leicester, Leicester, UK

^Deceased

Supplementary Table S1. Most common AEs in Phase II clinical trials of astegolimab[a]

	Astegolimab	Placebo
**ZENYATTA (severe asthma) [18]**	**n=375**	**n=127**
Asthma	142 (38)	60 (47)
Nasopharyngitis	50 (13)	14 (11)
Upper respiratory tract infection	23 (6)	12 (9)
Headache	29 (8)	6 (5)
Injection site reaction	24 (6)	1 (1)
Back pain	15 (4)	7 (6)
Rhinitis	12 (3)	7 (6)
**ZARNIE (moderate-to-severe atopic dermatitis) [35]**	**n=34**	**n=31**
Dermatitis atopic	3 (9)	5 (16)
Nasopharyngitis	0	3 (10)
Abdominal pain	0	2 (6)
**COPD-ST2OP (moderate-to-very-severe COPD) [19]**	**n=122 events**	**n=100 events**
Headache	23 (19)	11 (11)
Infections[b]	20 (16)	20 (20)
Gastrointestinal disorders[c]	20 (16)	14 (14)
**COVASTIL (severe COVID-19 pneumonia) [34]**	**n=130**	**n=134**
Constipation	10 (8)	6 (4)
Hypokalemia	9 (7)	8 (6)
Anemia	9 (7)	7 (5)
Hypotension	9 (7)	7 (5)
COVID-19 pneumonia	8 (6)	7 (5)
Acute kidney injury	5 (4)	7 (5)
Atrial fibrillation	8 (6)	2 (1)
Headache	7 (5)	4 (3)
Hypertension	8 (6)	5 (4)
Pneumothorax	3 (2)	7 (5)

All values are n (%).

[a]For ZENYATTA, ZARNIE, and COVASTIL, AEs occurring in ≥5% of patients in either treatment arm are reported; for COPD-ST2OP, the three most common AE categories as a proportion of total events are reported.

[b]Upper respiratory tract infection, lower respiratory tract infection, urinary tract infections, and cellulitis.

[c]Diarrhea, vomiting, and gastritis.

AE, adverse event; COPD, chronic obstructive pulmonary disease; COVID-19, coronavirus disease 2019.

Supplementary Table S2. MACE, infusion-related reactions, anaphylaxis, anaphylactoid, and hypersensitivity reactions in Phase II clinical trials of astegolimab

	ZENYATTA (severe asthma) [18]		ZARNIE (moderate-to-severe atopic dermatitis) [35]		COPD-ST2OP (moderate-to-very-severe COPD) [19]		COVASTIL (severe COVID-19 pneumonia) [34]	
	**Astegolimab pooled[a] (n=375)**	**Placebo (n=127)**	**Astegolimab 490 mg SC Q4W (n=34)**	**Placebo (n=31)**	**Astegolimab 490 mg SC Q4W (n=42)**	**Placebo (n=39)**	**Astegolimab 700 mg IV on D1 ± 350 mg IV on D15 (n=130)**	**Placebo (n=134)**
**Potential MACE[b]**	**2 (1)**	**1 (1)**	0	0	0	**1 (3)**	**4 (3)**	**2 (1)**
Tachycardia	0	1 (1)	0	0	0	0	0	0
Chest pain	1 (<1)	0	0	0	0	0	0	0
Atrial fibrillation	1 (<1)	0	0	0	0	0	1 (1)	0
Heart failure	0	0	0	0	0	1 (3)	0	0
Cardiac arrest	0	0	0	0	0	0	0	1 (1)
Acute myocardial infarction	0	0	0	0	0	0	1 (1)	1 (1)
Cardiac failure	0	0	0	0	0	0	1 (1)	0
Left ventricular failure	0	0	0	0	0	0	1 (1)	0
**Infusion-related reaction,[c] anaphylaxis, anaphylactoid, and hypersensitivity reaction, n (%)**	**1 (<1)**	**1 (1)**	**0**	**0**	**0**	**0**	**0[d]**	**2 (1)[d]**
Anaphylactic reaction	0	1 (1)	0	0	0	0	0[d]	0[d]
Hypersensitivity	1 (<1)	0	0	0	0	0	0[d]	0[d]
Tachycardia	0	0	0	0	0	0	0[d]	1 (1)[d]
Respiratory distress	0	0	0	0	0	0	0[d]	1 (1)[d]

All values are n (%).

[a]Pooled data for patients receiving astegolimab SC 70 mg Q4W (n=127), 210 mg Q4W (n=126), or 490 mg Q4W (n=122).

[b]Total number of MACE in COVASTIL was previously reported [34]; all other MACE data are data on file. MACE was not an AESI for ZENYATTA.

[c]Infusion-related reactions applicable to COVASTIL only. Anaphylaxis, anaphylactoid, and hypersensitivity reactions were not AESIs for ZENYATTA.

[d]Data on file.

AESI, adverse event of special interest; COPD, chronic obstructive pulmonary disease; COVID-19, coronavirus disease 2019; D, day; IV, intravenous; MACE, major cardiac adverse events; Q4W, every 4 weeks; SC, subcutaneous.
